# ^13^C-NMR Spectral Data of Alkaloids Isolated from *Psychotria* Species (Rubiaceae)

**DOI:** 10.3390/molecules22010103

**Published:** 2017-01-11

**Authors:** Almir Ribeiro de Carvalho Junior, Ivo Jose Curcino Vieira, Mario Geraldo de Carvalho, Raimundo Braz-Filho, Mary Anne S. Lima, Rafaela Oliveira Ferreira, Edmilson José Maria, Daniela Barros de Oliveira

**Affiliations:** 1 Laboratório de Ciências Químicas, CCT, Universidade Estadual do Norte Fluminense Darcy Ribeiro, Campos dos Goytacazes, RJ 28013-602, Brazil; almir@uenf.br (A.R.d.C.J.); braz@uenf.br (R.B.-F.); edmilson_maria@yahoo.com.br (E.J.M.); 2 Departamento de Química, ICE, Universidade Federal Rural do Rio de Janeiro, Seropédica, RJ 23890-000, Brazil; mgeraldo@ufrrj.br; 3 Departamento de Química Orgânica e Inorgânica, Centro de Ciências, Universidade Federal do Ceará, Fortaleza, CE 60021-940, Brazil; mary@dqoi.ufc.br; 4 Colegiado de Ciências Agrárias e Biotecnológicas, Universidade Federal do Tocantins, Gurupi, TO 77402-970, Brazil; rafaellaoliveira@mail.uft.edu.br; 5 Laboratório de Tecnologia de Alimentos, CCTA, Universidade Estadual do Norte Fluminense Darcy Ribeiro, Campos dos Goytacazes, RJ 28013-602, Brazil; dbarrosoliveira@uenf.br

**Keywords:** Rubiaceae, *Psychotria*, ^13^C-NMR spectral data

## Abstract

The genus *Psychotria* (Rubiaceae) comprises more than 2000 species, mainly found in tropical and subtropical forests. Several studies have been conducted concerning their chemical compositions, showing that this genus is a potential source of alkaloids. At least 70 indole alkaloids have been identified from this genus so far. This review aimed to compile ^13^C-NMR data of alkaloids isolated from the genus *Psychotria* as well as describe the main spectral features of different skeletons.

## 1. Introduction

In phytochemistry and related areas, structural elucidation techniques play a key role because precise knowledge of the chemistry of plants requires unequivocal structural characterization of its metabolites to obtain information related to the taxonomy of plant groups. Moreover, correct identification of biologically active compounds is important, both to understand their possible mechanisms of action and propose chemical modifications aimed at enhancing their activity.

The characterization of natural products requires, apart from patience and dedication, knowledge about spectroscopic techniques (interpretation of these data) and the biosynthesis of different types of metabolites. Comparison with literature data is another important auxiliary tool that aids the structural characterization of a given compound. In this context, finding a material that provides as much information as possible about the spectral data of metabolites isolated from a genus (such as *Psychotria*) may enable saving time.

The genus *Psychotria* (Rubiaceae) comprises more than 2000 species, which occur mostly in tropical and subtropical regions [1], with many of these species being employed in folk medicine to treat several diseases [2,3]. The biological potential of the chemical constituents of the species of this genus has possibly motivated several studies regarding the chemical composition of such species. Most of these have focused on investigating alkaloid fractions obtained by acid-base extraction, probably owing to the biological importance of this type of metabolite. Such efforts have led to the isolation and/or identification of various alkaloids, primarily indole-type. Some of them exhibit some biological properties such as analgesic [4,5], antioxidant [6], antiparasitic [7], and cytotoxic [8,9] activities. This review aimed to compile ^13^C-NMR spectral data of alkaloids isolated from *Psychotria* species as well as to discuss the main spectral features observed for the different types of skeletons.

## 2. Discussion

### 2.1. ^13^C-NMR Chemical Shifts of Monoterpene Indole Alkaloids Isolated from Psychotria Species

**Figure 1:**
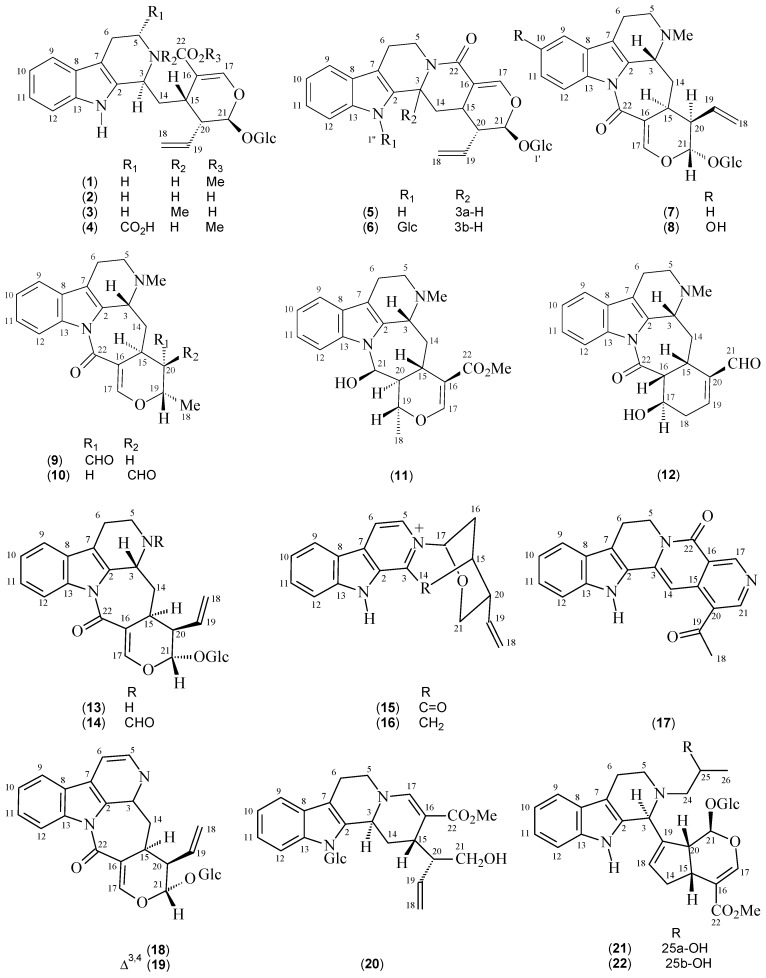

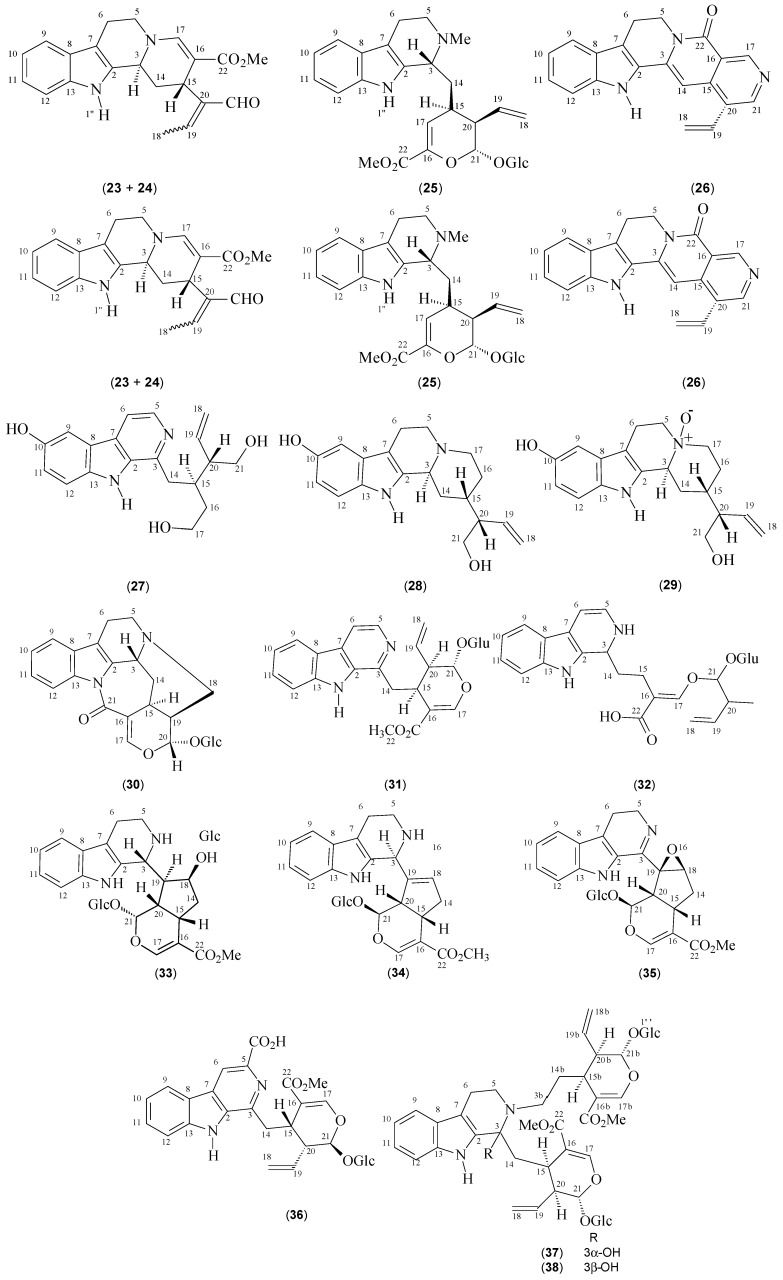
Structures of monoterpene indole alkaloids from *Psychotria* species.

Monoterpene indole alkaloids (MIAs) comprise a wide group of secondary metabolites, found mainly in the Apocynaceae, Loganiaceae, and Rubiaceae families [10]. Their biosynthesis involves a reaction between tryptamine (derived from tryptophan) and the iridoid secologanin, catalyzed by strictosidine synthase [11]. This initial step leads to the formation of strictosidine (**1**, Table 1, Figure 1), the key precursor of other MIAs.

Strictosidine (**1**) was isolated from *P. elata* [12] and *P. nuda* (data not reported), and presents an *ortho*-substituted ring system (as do most of the MIAs isolated from this genus), characterized by the presence of four methine carbon signals at *δ*_C_ 118.8 (CH-9), 120.1 (CH-10), 122.7 (CH-11), and 112.0 (CH-12), and two quaternary carbon signals at *δ*_C_ 127.9 (C-8) and 137.9 (C-13). The signals of two quaternary carbons at *δ*_C_ 133.2 (C-2) and 107.7 (C-7), along with a methine carbon at *δ*_C_ 52.4 (CH-3) and two methylene carbons at *δ*_C_ 42.9 (CH_2_-5) and 21.0 (CH_2_-6) complete the tetrahydro-β-carboline system. The secologanin moiety is confirmed by the presence of signals resonating at *δ*_C_ 170.6 (C-22), *δ*_C_ 109.9 (C-16), and 156.1 (C-17), indicative of an α,β-unsaturated carboxyl group, a terminal vinyl at *δ*_C_ 135.7 (CH-19) and 119.5 (CH_2_-18), besides signals at *δ*_C_ 97.5 (CH-21), 45.6 (CH-20), 35.9 (CH_2_-14), and 32.5 (CH-15). The anomeric carbon signals of the glucose unit are observed at *δ*_C_ 100.3 (CH-1’), with four mono-oxygenated methines in the interval from *δ*_C_ 78.6 to 71.7 and one oxygenated methylene at *δ*_C_ 62.9 [13].

Strictosidine (**1**) may function as a precursor of other biosynthetic pathways, leading to different skeletons and consequently changes in spectral properties. Carbonylation at C-5 (*δ*_C_ = 176.5 ppm), as observed for 5*α*-carboxystrictosidine (**4**), for example, promotes a chemical shift displacement of CH_2_-6 (Δ*δ* = 4.2 ppm, *β* effect) when compared with **1**, as can be seen in Table 2. A similar pattern was observed for methylation of *N*-4 on correantoside (**7**) isolated from *P. correa* [14], where Δ*δ* variations (*β* effect) of 5.4 and 3.5 ppm are observed for CH-3 and CH_2_-5, respectively. For 10-hydroxycorreantoside (**8**), it is possible to observe the electronic influence of a hydroxyl by the inductive effect at the *ipso* carbon (C-10) and an increase in the electron densities at the *ortho*(CH-9 and CH-11) and *para* (C-13) positions by the mesomeric effect. On the basis of this mesomeric effect, the signals corresponding to carbon atoms at the *ortho,* CH-9 (*δ*_C_ 118.8 (**1**) and 104.4 (**8**), Δ*δ*_C_ = −14.8 ppm) and CH-11 (*δ*_C_ 122.7 (**1**) and 114.2 (**8**), Δ*δ*_C_ = −8.5 ppm) and *para* positions, C-13 (*δ*_C_ 137.9 (**1**) and 131.4 (**8**), Δ*δ*_C_ = −6.5 ppm), are displaced upfield.

**Figure 2:**
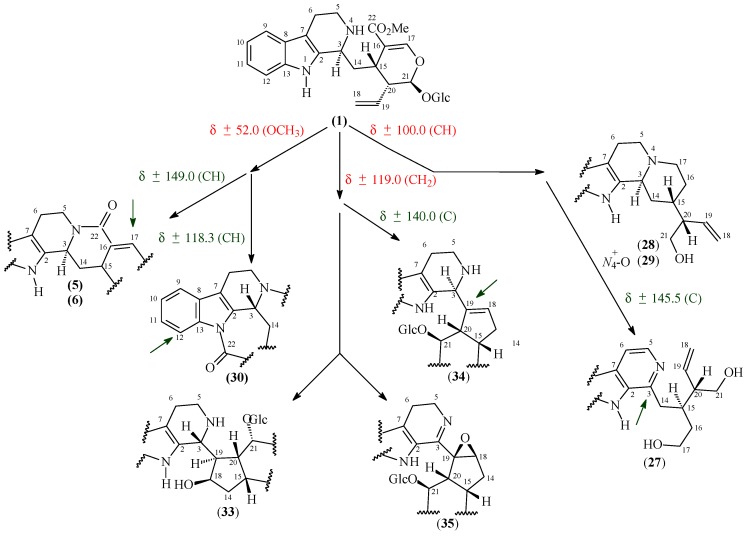
Structural signaling based on specific signals compared with values for strictosidine (**1**): absence of a given signal in red and presence in green.

Other metabolic pathways of this class of alkaloid revealed cyclization reactions involving *N*-1(compounds **5** and **6**) or *N*-4 (compounds **7**–**14**) with C-22, or *N*-1 with C-18 and *N*-4 with C-22, as particularly observed for stachyoside (**30**) isolated from *P. stachyoides* [15] (Figure 2). Strictosamide (**5**), isolated from four different species, [16,17,18,19] is an example of lactam formation between *N*-4 and C-22. By examining Table 2, it is possible to notice, apart from the absence of a methoxyl group (carbomethoxy function) signal at *δ*_C_ 52.4, a slight difference in the chemical shift of C-22 (*δ*_C_ 167.1 ppm), when compared with compound **1** (*δ*_C_ 170.6 ppm), as well as a Δ*δ*_C_ variation of 6.9 ppm for C-17. In contrast, correantoside (**7**) exemplified the first possibility involving cyclization between *N*-1 and C-22. It is possible, in this case, to observe the variation in the chemical shifts of the *ortho*CH-12 (Δ*δ* = 4.0 ppm) and *para* CH-10 (Δ*δ* = 4.1 ppm) atoms, promoted by the inductive and mesomeric effects of the carboxyl group at C-22. These effects were also observed for compounds **8** (Δ*δ*_C_ = 4.8 (CH-12) ppm), **13** (Δ*δ*_C_ = 4.4 (CH-12) and 4.1 (CH-10) ppm), **14** (Δ*δ*_C_ = 4.4 (CH-12) and 4.5 (CH-10) ppm), **18** (Δ*δ*_C_ = 7.8 (CH-12) and 6.2 (CH-10) ppm), and **19** (Δ*δ*_C_ = 7.3 (CH-12) and 5.4 (CH-10) ppm), showing that the downfield displacements of the CH-12 and CH-10 signals may be used to suggest that *N*-1 is attached to C-22.

There are some examples of alkaloids isolated from this genus, whose biosynthesis involves hydrolysis of a glycoside moiety such as (*E*/*Z*)-vallesiachotamines, **23** and **24**, isolated from *P. bahiensis* [17], and10-hydroxy-*iso*-deppeaninol (**27**) and *N*-oxide-10-hydroxyantirhine (**29**) isolated from *P. prunifolia* [20]. These types of skeletons may be suggested by analysis of the region of the ^13^C spectrum that is typical of sugars, revealing the absence of the typical signal of the anomeric carbon around *δ*_C_ 100.0, apart from additional signals of the oxy-carbons characteristic of this unit.

Kerber et al. reported the isolation of a new MIA from *P. brachyceras* leaves [21], named brachycerine (**33**), which showed a new alkaloid skeleton. Its biosynthesis involved the coupling of tryptamine to a 1-*epi*-loganin derivative. Psychollatine (**34**), a new MIA from *P. umbellate* [22], presented a terpenoid derivative from geniposide. Both alkaloids as well as compounds **21**, **22**, and **35** revealed an important characteristic in their ^13^C spectra: the absence of typical signals of a terminal vinyl group (~*δ*_C_ 119 ppm). In contrast, bahienosides A (**38**) and B (**37**), isolated from *P. bahiensis* [17], showed duplicate terminal vinyl group signals relative to two secologanin moieties. Figure 2 shows typical carbon assignments, which may indicate some different structural possibilities in comparison with those values observed for strictosidine (**1**).

### 2.2. ^13^C-NMR Chemical Shifts of Pyrrolidinoindoline Alkaloids Isolated from Psychotria Species

**Figure 3:**
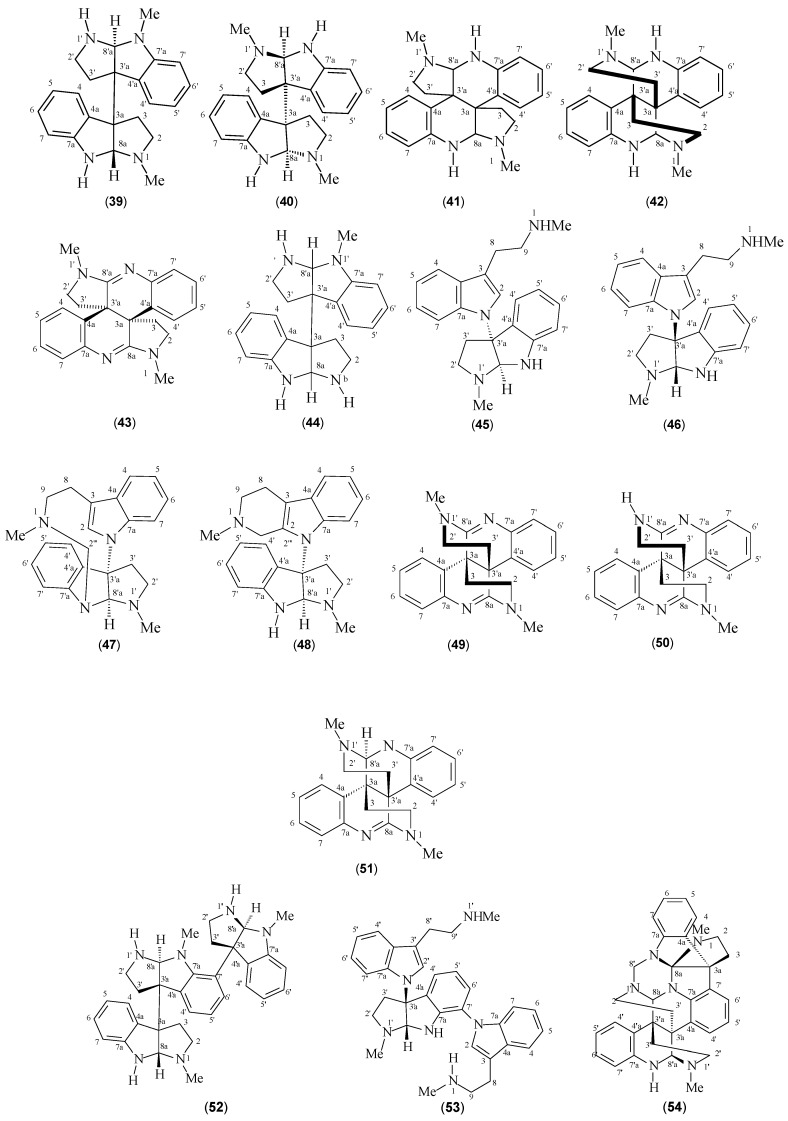

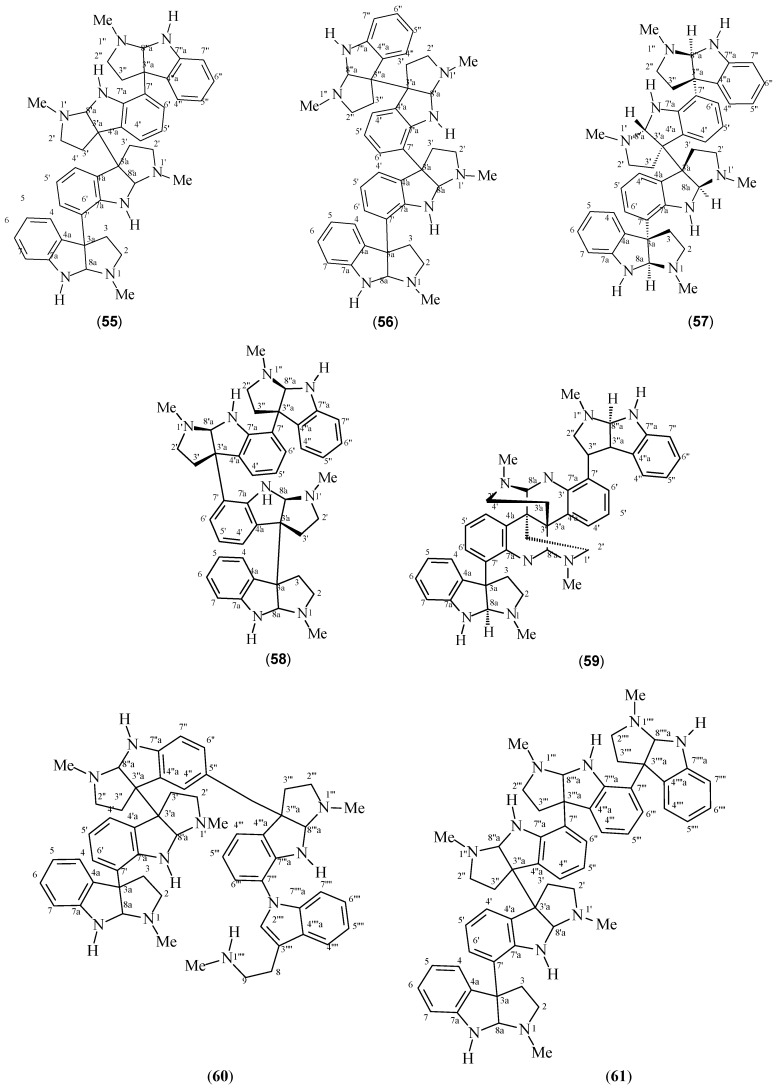

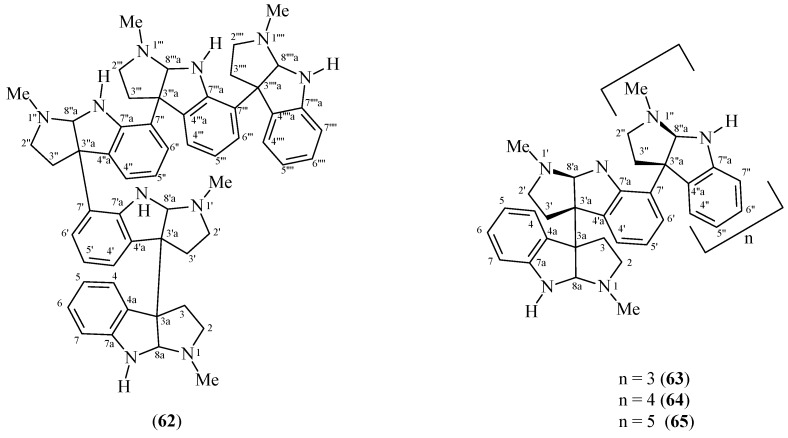
Structures of pyrrolidinoindoline alkaloids from *Psychotria* species.

Some studies have also reported that the isolation of pyrrolidinoindoline alkaloids seems to be specific to the *Psychotria* species (Table 3). As shown in Figure 3, their chemical structures present the condensation of some *N*-methyltryptamine units with different connection patterns, mainly involving C-3a-C3’a, C-3’a-C-7, and *N*-C-3’a bonds or containing *N*-methyltryptamine units linked to a bisquinoline part. The compound (+)-chimonanthine (**40**) was isolated from several *Psychotria* species [40,41,42] and is an example of a dimer that presents a C-3a-C-3’a-type linkage between its two units. Its ^13^C-NMR spectrum exhibited 11 carbon-signal equivalents for both units. The signals at *δ*_C_ 52.4 (CH_2_-2) and 84.6 (C-8a) are typical of carbons bearing one and two nitrogen atoms, respectively. The signals at *δ*_C_ 33.2 and 63.6 were attributed to C-3 and C-3a, respectively, whereas the signal at *δ*_C_ 33.8 is consistent with a methyl carbon attached to a nitrogen atom. The *ortho*-substituted aromatic rings are characterized by signals at *δ*_C_ 124.9 (CH-4/CH-4’), 128.3 (C-4a/C-4a’), 122.3 (CH-5), 119.8 (CH-5’), 129.9 (CH-6/CH-6’), 110.5 (CH-7/CH-7’), and 150.5 (C-7a/C-7a’) [40] (Table 4).

Since some compounds with more than two units present a chimonanthine portion in their structures, the monitoring of C-3a and C-7 (main binding sites) and their neighborhood may be a good alternative, in order to determine the positions of the other monomeric units. Hodgkinsine (**52**) occurs frequently in the genus [41,42,43,44,45,46] and presents a third unit with a C-3’’a-C-7’ linkage. In this case, besides replacement of a methine aromatic carbon by a quaternary carbon (C-7’), observing the up field displacements of C-6’ and C-4’ (Δ*δ* around 3.0 ppm) is possible probably because of the presence of a group that increases the electron densities of these positions (comparison with compound **40**). Takayama et al. (2004), however, reported the isolation of psychopentamine (**60**) from *P. rostrata*^2^, which showed a new type of linkage between C-3’’’a and C-5’’ [2].

The chemical study of *P. calocarpa* leaves [43] led to the isolation of a new alkaloid named psychotriasine (**45**), which presents a tryptamine unit linked to a pyrroloindole unit by an *N*-C3’a linkage. This type of junction was also observed for psychohenin (**46**) and compound **48** isolated from *P. henryi* [47,48] and may be indicated by the presence of a quaternary carbon (C-3’a) that resonates at *δ*_C_ 79.4, 77.8, and 76.7 ppm, in the three compounds, respectively. In contrast, psychotrimine (**53**), isolated from *P. rostrate* [2] shows, besides the *N*-C-3’a bond, an *N*-C-7’ linkage indicated by the signal of a quaternary aromatic carbon C-7’ at *δ*_C_ 121.5 ppm.

Alkaloids with more complex structures, containing from four to seven units, such as quadrigemines A–C (**55**–**57**), psychotridine (**61**), oleoidine (**64**), and caledonine (**65**), have also been isolated from this genus; however, the structural elucidation of these compounds becomes more difficult as the number of units increases. Probably owing to this, some studies did not provide detailed attributions of their carbon signals. In such cases, mass spectrometry plays an important role in establishing the number of units present in their structures as well as the pattern of the junctions.

### 2.3. ^13^C-NMR Chemical Shifts of Benzoquinolizidine Alkaloids Isolated from Psychotria Species

Muhammad et al. reported the isolation of five benzoquinolizidine alkaloids from *Psychotria klugii* [7] (Table 5). Among them, klugine (**66**) and 7’-*O*-demethylisocephaeline (**67**) were reported for the first time, whereas cephaeline (**68**), isocephaeline (**69**), and 7-*O*-methylipecoside (**70**) were previously isolated from *Cephaelis* species [56,57].

**Figure 4:**
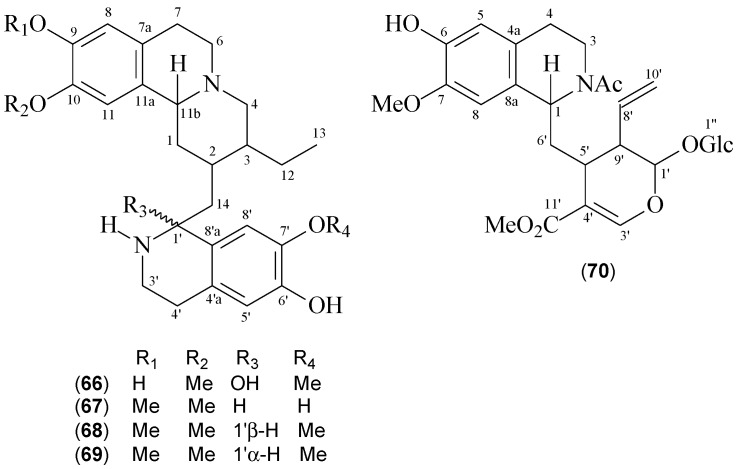
Structures of Benzoquinolizidine Alkaloids from *P. klugii.*

Compound **68** (ipecac alkaloid) as along with compounds **66**, **67**, and **69** possesses an unusual skeleton with two tetrahydroisoquinoline ring systems [10] characterized by the presence of four quaternary carbon signs at *δ*_C_ 147.2, 147.5 (C-9 and C-10, oxygenated *ortho*-substituted carbons), 126.8 (C-7a), and 130.1 (C-11a), two methine carbons at *δ*_C_ 108.6 (CH-11) and 111.5 (C-8), and signals at *δ*_C_ 62.4 (CH-11b), 52.3 (CH_2_-6), and 29.2 (CH_2_-7). A similar system is observed for the lower unit, with the exception of the absence of a methoxyl group attaching C-6’ (a hydroxyl group in this position). The remarkable difference between compounds **68** and **69** (stereoisomers) is associated with the chemical shift of carbon C-1′ at *δ*_C_ 51.9 and 55.3 respectively, whereas compounds **66** and **67** differ from **68** and **69** in the number and positions of the methoxyl groups. Interestingly, compound **70** exhibits carbon assignments consistent with a tetrahydroisoquinoline ring attached to a secologanin moiety at C-1.The chemical structures of compounds **68**–**70** are shown in Figure 4, and their ^13^C-NMR data are listed in Table 6.

## 3. Conclusions

In this work, we attempted to compile ^13^C-NMR data of alkaloids isolated from the *Psychotria* genus and provide information that may be useful in order to distinguish different types of skeletons. For monoterpene indole alkaloids (MIAs), mainly found in tropical species, a good strategy for their structural elucidation is to compare their spectral data with those observed for strictosidine (**1**). The monitoring of differences in specific parts of the spectrum, such as the signals of C-22, CH-17, CH-12, CH_2_-5, and CH-1′, may suggest alternative structural possibilities. Note that all comparisons performed in this work are restricted where possible to compounds whose ^13^C-NMR experiments were run in the same solvent.

The main pyrrolidinoindoline alkaloids found in this genus are chimonanthine derivatives, with units linked mostly by C3a-C3’a or C-3a-C7a bonds. Some examples have shown different patterns of linkages between *N* (from tryptamine terminal units) and C-3a. For compounds with more than three units, such as quadrigemines A–C and psychotridine and its isomer, obtaining detailed assignments of these carbons is not possible owing to structural complexity.The occurrence of benzoquinolizidine alkaloids in *Psychotria* species is less common, comprising some compounds isolated from *Psychotria klugii*.

## Figures and Tables

**Table 1:** Monoterpene indole alkaloids from *Psychotria* species.

Compounds	Species	References	^13^C-NMR Data
Strictosidine (**1**)	*P. elata*	[12]	[13]
Strictosidinic acid (**2**)	*P. acuminata* *P. barbiflora* *P. myriantha*	[1,23,24,25]	[25]
Palicoside (**3**)	*P. racemosa*	[12]	[26]
5α-Carboxystrictosidine (**4**)	*P. acuminata* *P. bahiensis*	[17,23]	[27]
Strictosamide (**5**)	*P. bahiensis* *P. nuda* *P. prunifolia* *P. suterella*	[16,17,18,19]	[18]
*N*,*β*-D-Glucopyranosilvincosamide (**6**)	*P. leiocarpa*	[28]	[28]
Correantoside (**7**)	*P. correae*	[14]	[14]
10-Hydroxycorreantoside (**8**)	*P. correae*	[14]	[14]
Correantine B (**9**)	*P. correae*	[14]	[14]
20-*epi*-Correantine B (**10**)	*P. correae*	[14]	[14]
Correantine A (**11**)	*P. correae*	[14]	[14]
Correantine C (**12**)	*P. correae*	[14]	[14]
*N*-Desmethyl-correantoside (**13**)	*P. stachyoides*	[29]	[29]
*Nor*-Methyl-23-oxo-correantoside (**14**)	*P. stachyoides*	[15]	[15]
14-Oxoprunifoleine (**15**)	*P. prunifolia*	[18,20]	[18]
17-Vinyl-19-oxa-2-azonia-12-azapentacyclo[14.3.1.0^2,14^.0^5,13^.0^6,11^]icosa-2(14),3,5(13),6(11),7,9-hexaene (**16**)	*P. prunifolia*	[18]	[18]
Naucletine (**17**)	*P. suterella*	[19]	[30]
Correantosine E (**18**)	*P. stachyoides*	[31]	[31]
Correantosine F (**19**)	*P. stachyoides*	[31]	[31]
Lagamboside (**20**)	*P. acuminata*	[23]	[23]
*N*^4^-[1-((*R*)-2-Hydroxypropyl)]-psychollatine (**21**)	*P. umbellata*	[32]	[32]
*N*^4^-[1-((*S*)-2-Hydroxypropyl)]-psychollatine (**22**)	*P. umbellata*	[32]	[32]
(*E/Z*)-Vallesiachotamine (**23 + 24**)	*P. bahiensis* *P. laciniata*	[17,33]	[34]
Isodolichantoside (**25**)	*P. correae*	[14]	[14]
Angustine (**26**)	*P. bahiensis* *P. laciniata*	[17,33]	[35]
10-Hydroxy-*iso*-deppeaninol (**27**)	*P. prunifolia*	[20]	[20]
10-Hydroxy-antirhine (**28**)	*P. prunifolia*	[20]	[20]
*N*-Oxide-10-hydroxyantirhine (**29**)	*P. prunifolia*	[20]	[20]
Stachyoside (**30**)	*P. stachyoides*	[15]	[15]
Lyaloside (**31**)	*P. laciniata* *P. suterella*	[19,36]	[37]
Myrianthosine (**32**)	*P. myriantha*	[25]	[25]
Brachycerine (**33**)	*P. brachyceras*	[21]	[21]
Psychollatine (**34**)	*P. umbellata* *P. umbellata*	[5,22,38]	[22]
3,4-Dehydro-18,19-β-epoxy-psychollatine (**35**)	*P. umbellata*	[32]	[32]
Desoxycordifoline (**36**)	*P. acuminata*	[23]	[39]
Bahienoside B (**37**)	*P. acuminata* *P. bahiensis*	[17,23]	[17]
Bahienoside A (**38**)	*P. bahiensis*	[17]	[17]

**Table 2:** ^13^C-NMR data of MIAs from *Psychotria* species.

**Carbons**	**Compounds/*δ*_C_ (ppm)**									
	**1 ^I^**	**2 ^III^**	**3 ^III^**	**4 ^I^**	**5 ^I^**	**6 ^I^**	**7 ^I^**	**8 ^I^**	**9 ^II^**	**10 ^II^**
**C**										
**2**	133.2	132.3	134.7	133.2	134.8	136.1	134.3	133.8	132.9	133.0
**7**	107.7	106.0	105.2	109.0	110.3	111.5	115.7	115.3	114.8	114.8
**8**	127.9	126.1	126.6	128.0	128.7	129.5	130.4	131.3	129.1	129.1
**13**	137.9	135.8	135.8	138.4	137.8	137.7	137.3	131.4	136.0	136.0
**22**	170.6	170.0	168.4	170.9	167.1	166.3	168.2	167.8	166.2	166.2
**16**	109.9	113.4	112.5	109.9	109.2	109.1	112.2	112.0	108.6	109.6
**CH**										
**3**	52.4	49.6	56.1	53.2	55.1	54.5	57.8	58.2	56.4	56.7
**5**	-	-	-	60.1	-	-	-	-	-	-
**9**	118.8	117.8	117.4	118.8	118.7	119.3	119.2	104.4	118.1	118.0
**10**	120.1	118.7	118.1	120.1	120.2	121.3	124.2	155.1	123.2	123.2
**11**	122.7	121.2	120.3	122.6	122.6	122.9	125.5	114.2	124.6	124.6
**12**	112.0	111.5	110.8	112.1	112.3	114.8	116.0	116.8	115.4	115.2
**15**	32.5	31.8	30.6	32.4	24.9	27.9	35.7	35.6	29.7	29.2
**17**	156.1	150.0	151.8	156.1	149.2	149.2	155.7	155.5	158.0	156.4
**19**	135.7	135.6	135.6	135.2	134.4	133.4	135.1	135.0	70.2	69.4
**20**	45.6	44.3	44.0	45.7	44.7	44.1	45.4	45.4	51.8	53.9
**21**	97.5	95.1	95.9	97.6	98.1	97.5	97.4	97.3	-	-
**CH_2_**										
**5**	42.9	40.0	45.2	60.1	44.8	41.6	46.4	46.7	45.5	45.5
**6**	21.0	19.2	15.9	25.2	22.1	22.3	18.8	18.8	17.6	17.7
**14**	35.9	33.7	35.3	35.6	27.3	35.6	34.4	34.1	39.1	35.3
**18**	119.5	117.8	117.8	119.6	120.6	120.7	119.2	119.3	-	-
**CH_3_**										
**MeN-**	-	-	39.8	-	-	-	41.4	41.2	-	41.5
**Me**	-	-	-	-	-	-	-	-	18.3	19.3
**Glucose**										
**1′**	100.3	98.9	98.7	100.5	100.5	99.6	100.5	100.5	-	-
**2′**	78.6	69.8	73.0	74.7	74.3	74.9	74.7	74.7	-	-
**3′**	78.0	73.1	77.2	78.0	77.9	77.9	78.6	78.6	-	-
**4′**	74.6	77.2	70.0	71.9	71.3	71.6 ^a^	71.6	71.7	-	-
**5′**	71.7	76.5	76.6	78.6	78.2	78.3	78.0	78.0	-	-
**6′**	62.9	61.0	61.0	63.1	62.6	62.7	62.9	62.9	-	-
**1″**	-	-	-	-	-	87.6	-	-	-	-
**2″**	-	-	-	-	-	71.9	-	-	-	-
**3″**	-	-	-	-	-	75.1	-	-	-	-
**4″**	-	-	-	-	-	71.6 ^a^	-	-	-	-
**5″**	-	-	-	-	-	81.2	-	-	-	-
**6″**	-	-	-	-	-	62.9	-	-	-	-
**CHO**	-	-	-	-	-	-	-	-	199.5	199.2
**CO_2_Me**	52.4	-	-	52.6	-	-	-	-	-	-
**CO_2_H**	-	-	-	176.5	-	-	-	-	-	-
**Carbons**	**Compounds/*δ*_C_ (ppm)**									
	**11 ^II^**	**12 ^I^**	**13 ^I^**	**14 ^I^**	**15 ^I^**	**16 ^I^**	**17 ^II^**	**18 ^I^**	**19 ^I^**	**20 ^I^**
**C**										
**2**	136.2	134.6	136.0	132.4	134.4	132.2	127.4	145.7	134.4	136.0
**3**	-	-	-	-	139.7	139.5	140.8	-	148.1	-
**7**	108.0	117.4	117.0	116.2	124.6	132.9	116.9	138.0	134.0	111.3
**8**	126.8	130.6	131.0	130.1	118.9	119.7	125.7	123.9	125.2	129.7
**13**	137.1	137.7	137.3	137.7	146.9	144.6	139.0	140.0	142.3	136.0
**14**	-	-	-	-	191.6	-	-	-	-	-
**15**	-	-	-	-	-	-	141.1	-	-	-
**16**	111.2	-	112.7	111.8	-	-	117.1	113.5	114.5	95.3
**19**	-	-	-	-	-	-	199.6	-	-	-
**20**	-	-	-	-	-	-	138.8	-	-	-
**21**	-	194.3	-	-	-	-	-	-	-	-
**22**	167.5	174.8	168.6	168.1	-	-	161.6	167.9	168.8	171.8
**CH**										
**3**	61.4	58.5	50.6	47.9	-	-	-	50.0	-	50.2
**5**	-	-	-	-	134.1	132.5	-	137.0	142.6	-
**6**	-	-	-	-	120.6	116.0	-	116.2	114.5	-
**9**	118.5	117.8	119.2	119.5	123.6	122.8	119.3	123.9	122.3	119.0
**10**	119.8	119.0	124.4	124.6	123.4	122.4	119.9	126.3	125.5	121.0
**11**	121.5	125.0	125.5	126.0	137.2	132.3	120.9	133.5	131.3	122.7
**12**	109.2	126.0	116.4	116.4	113.7	113.2	112.0	119.8	119.3	114.6
**14**	-	-	-	-	-	-	95.6	-	-	-
**15**	30.8	34.5	35.7	35.6	42.8	25.6	-	30.5	21.2	33.7
**16**	-	52.0	-	-	-	-	-	-	-	-
**17**	155.2	67.5	155.6	156.5	87.9	86.7	154.0	157.2	155.9	149.3
**19**	74.8	149.7	135.2	134.9	132.8	134.9	-	133.6	134.1	140.8
**20**	52.0	-	45.6	45.3	42.0	41.2	-	46.4	46.7	55.2
**21**	75.5	-	97.5	97.6	-	-	155.4	97.9	97.9	-
**CH_2_**										
**5**	52.0	48.0	40.0	41.6	-	-	40.7	-	-	53.0
**6**	20.9	19.6	23.2	23.2	-	-	19.8	-	-	23.4
**14**	36.7	35.9	36.7	34.8	-	24.8	-	36.7	39.8	35.1
**16**	-	-	-	-	42.8	25.6	-	-	-	-
**18**	-	33.8	119.3	119.5	118.9	117.9	-	121.8	121.3	116.9
**21**	-	-	-	-	63.4	61.9	-	-	-	65.4
**CH_3_**										
**18**	18.6	-	-	-	-	-	29.3	-	-	-
**MeN-**	43.0	41.9	-	-	-	-	-	-	-	-
**Glucose**										
**1′**	-	-	100.7	100.8	-	-	-	100.1	100.1	87.6
**2′**	-	-	74.9	74.9	-	-	-	74.8	74.7	72.4
**3′**	-	-	78.7	78.2	-	-	-	78.0	77.9	79.4
**4′**	-	-	71.8	71.7	-	-	-	71.7	71.7	71.8
**5′**	-	-	78.2	78.7	-	-	-	78.0	78.6	81.2
**6′**	-	-	63.1	63.0	-	-	-	62.9	62.9	63.0
**CHO**	-	-	-	163.9	-	-	-	-	-	-
**CO_2_Me**	51.1	-	-	-	-	-	-	-	-	51.2
**Carbons**	**Compounds/*δ*_C_ (ppm)**									
	**21 ^I^**	**22 ^I^**	**23 ^III^**	**24 ^III^**	**25 ^I^**	**26 ^III^**	**27 ^I^**	**28 ^I^**	**29 ^I^**	
**C**										
**2**	134.0	133.4	133.1	133.6	134.0	126.8	136.9	130.5	131.0	
**3**	-	-	-	-	-	136.9	145.5	-	-	
**7**	108.4	108.4	106.6	107.4	106.5	114.8	130.6	106.0	105.7	
**8**	138.6	138.1	126.2	127.0	128.1	125.5	123.1	128.6	128.3	
**10**	-	-	-	-	-	-	152.6	151.8	152.0	
**13**	128.4	128.0	136.1	136.8	137.8	138.5	137.5	133.1	133.6	
**15**	-	-	-	-	-	139.0	-	-	-	
**16**	112.2	112.0	93.2	93.4	112.0	119.8	-	-	-	
**19**	141.0	142.1	-	-	-	-	-	-	-	
**20**	-	-	146.1	143.9	-	127.8	-	-	-	
**22**	169.7	169.0	166.9	167.6	169.8	161.1	-	-	-	
**CH**										
**3**	61.7	59.3	48.6	47.9	58.8	-	-	57.0	71.6	
**5**	-	-	-	-	-	-	135.7	-	-	
**6**	-	-	-	-	-	-	114.6	-	-	
**9**	118.6	118.0	117.4	118.4	118.7	119.9	106.6	103.2	103.3	
**10**	119.5	120.0	118.3	119.2	119.9	119.9	-	-	-	
**11**	121.9	122.0	120.7	121.6	122.3	124.6	120.4	112.9	113.2	
**12**	112.0	112.0	110.8	111.8	111.8	112.0	113.7	112.8	113.0	
**14**	-	-	-	-	-	93.8	-	-	-	
**15**	33.0	35.3	27.4	30.5	30.5	-	36.4	31.1	30.6	
**17**	153.3	153.0	147.2	148.5	154.0	149.7	-	-	-	
**18**	132.6	131.0	-	-	-	-	-	-	-	
**19**	-	-	152.0	146.3	135.8	130.2	138.1	138.7	138.2	
**20**	49.0	48.4	-	-	45.5	-	51.0	50.8	52.3	
**21**	95.6	97.0	-	-	97.8	147.7	-	-	-	
**25**	65.1	66.3	-	-	-	-	-	-	-	
**CH_2_**										
**5**	49.0	49.6	49.8	50.7	47.9	40.4	-	52.4	69.0	
**6**	21.4	19.7	21.3	22.2	17.9	19.2	-	18.1	20.6	
**14**	39.4	39.5	32.9	32.9	34.5	-	37.0	31.6	28.5	
**17**	-	-	-	-	-	-	61.4	48.0	59.1	
**18**	-	-	-	-	119.8	119.8	118.7	118.5	118.5	
**21**	-	-	-	-	-	-	64.4	64.0	63.8	
**24**	62.4	61.6	-	-	-	-	-	-	-	
**CH_3_**										
**18**	-	-	14.3	13.8	-	-	-	-	-	
**26**	20.7	21.2	-	-	-	-	-	-	-	
**MeN-**	-	-	-	-	40.6	-	-	-	-	
**Glucose**										
**1′**	100.1	100.1	-	-	100.5	-	-	-	-	
**2′**	74.6	74.8	-	-	74.7	-	-	-	-	
**3′**	78.0	78.0	-	-	78.6	-	-	-	-	
**4′**	78.2	71.6	-	-	71.6	-	-	-	-	
**5′**	76.2	78.5	-	-	78.0	-	-	-	-	
**6′**	62.7	62.5	-	-	62.9	-	-	-	-	
**CHO**	-	-	195.5	191.5	-	-		-	-	
**CO_2_Me**	51.6	51.7	49.7	50.8	51.9	-	-	-	-	
**Carbons**				**Compounds/*δ*_C_ (ppm)**						
	**30 ^I^**	**31**	**32 ^III^**	**33 ^I^**	**34 ^I^**	**35 ^I^**	**36 ^I^**	**37 ^I^**	**38 ^I^**	
**C**										
**2**	137.1	140.3	134.8	130.7	131.1	128.8	135.6	135.0	138.0	
**3**	-	143.8	-	-	-	158.9	142.9	-	-	
**5**	-	-	-	-	-	-	135.6	-	-	
**7**	118.4	121.0	121.0	108.3	107.9	118.8	128.4	107.3	106.6	
**8**	129.2	126.9	121.5	127.7	127.6	139.9	121.7	128.4	128.0	
**13**	139.1	134.6	140.2	112.3	138.1	126.1	141.6	137.8	138.0	
**15**										
**19**	-	-	-	-	140.0	67.3	-	-	-	
**16**	114.8	109.9	112.0	111.8	112.2	110.2	108.7	112.1	111.5	
**21**	169.0	-	-	-	-	-	-	-	-	
**22**	-	166.6	170.0	169.1	169.1	168.9	171.3	169.7	170.0	
**22b**	-	-		-	-	-	-	169.5	169.4	
**CH**										
**3**	51.7	-	48.5	54.7	53.7	-	-	58.8	59.6	
**5**	-	137.3	137.0	-	-	-	-	-	-	
**6**	-	112.6	118.0	-	-	-	114.2	-	-	
**9**	119.7	121.4	126.6	118.9	119.0	121.2	121.4	120.6	118.7	
**10**	125.2	119.0	118.9	120.2	120.3	121.1	119.9	119.7	120.0	
**11**	126.8	127.6	127.5	123.2	123.6	126.2	128.4	122.0	122.5	
**12**	118.3	111.8	112.5	112.3	112.3	113.6	111.6	112.0	112.0	
**15**	32.9	30.1	-	35.5	37.5	31.7	34.5	31.5	31.6	
**17**	148.7	151.6	151.0	153.5	153.4	153.1	153.2	154.0	154.7	
**18**	-	-	-	74.3	138.5	62.5	-	-	-	
**19**	53.3	134.0	134.5	49.0	-	-	133.8	136.2	136.1	
**20**	95.5	42.9	45.5	41.9	49.0	43.8	44.4	45.5	45.4	
**21**	-	95.9	95.4	99.0	99.4	95.2	96.1	98.2	97.9	
**15b**	-	-		-	-	-	-	30.3	30.5	
**17b**	-	-		-	-	-	-	153.2	153.5	
**19b**	-	-		-	-	-	-	135.7	135.5	
**20b**	-	-		-	-	-	-	44.8	44.8	
**21b**	-	-		-	-	-	-	98.5	98.3	
**CH_2_**										
**5**	48.0	-	-	41.8	42.1	48.2	-	44.8	44.8	
**6**	21.2	-	-	24.4	20.5	20.1	-	17.6	17.4	
**14**	43.7	32.1	45.6	43.5	40.5	34.7	34.0	36.9	36.7	
**15**	-	-	30.0	-	-	-	-	-	-	
**17**	-	-	-	-	-	-	-	-	-	
**18**	72.4	118.6	118.9	-	-	-	117.6	119.8	119.8	
**3b**	-	-		-	-	-	-	52.0	51.9	
**14b**	-	-	-	-	-	-	-	28.0	27.4	
**18b**	-	-	-	-	-	-	-	120.1	120.1	
**CH_3_**										
**Me**	-	-	10.4	-	-	-	-	-	-	
**Glucose**										
**1′**	100.3	98.79	98.6	100.6	101.5	99.4	99.0	100.4	100.4	
**2′**	74.9	73.1	73.0	74.0	71.0	74.7	73.2	74.6	74.8 ^c^	
**3′**	78.5	77.3	69.9	71.1	78.6	78.1	76.6	78.0	78.6 ^a^	
**4′**	71.9	71.10	77.3	78.3	74.6	72.1	70.4	71.6	71.7 ^d^	
**5′**	78.5	77.8	76.8	77.7	77.6	78.8	76.6	78.4	78.2 ^b^	
**6′**	63.0	61.2	61.0	62.1	61.8	63.3	61.8	62.9	62.9 ^e^	
**1″**	-	-	-	-	-	-	-	100.3	100.4	
**2″**	-	-	-	-	-	-	-	74.8	74.6 ^c^	
**3″**	-	-	-	-	-	-	-	78.1	78.4 ^a^	
**4″**	-	-	-	-	-	-	-	71.6	71.6 ^d^	
**5″**	-	-	-	-	-	-	-	78.3	78.0 ^b^	
**6″**	-	-	-	-	-	-	-	62.8	62.8 ^e^	
**CO_2_Me**	-	50.7	-	51.8	51.9	51.9	50.6	52.1	52.1	

^I^ CD_3_OD, ^II^ CDCl_3_ e ^III^ DMSO-*d*_6_, letters (a–e) indicate signals that may be interchanged.

**Table 3:** Pyrrolidinoindoline alkaloids from *Psychotria* species.

Compounds	Species	References	^13^C-NMR Data
*Meso*-chimonanthine (39)	*P. forsteriana* *P. muscosa*	[41,49,50]	[50]
(+)-Chimonanthine (**40**)	*P. colorata* *P. muscosa* *P. rostrata* *P. hoffmannseggiana*	[40,41,42]	[40]
*Iso*-calycanthine (41)	*P. forsteriana*	[50]	[50]
Calycanthine (**42**)	*P. forsteriana*	[50]	[50]
(8-8a),(8’-8’a)-tetradehydroisocalycanthine 3a(*R*), 3’a(*R*) (**43**)	*P. colorata*	[42]	[42]
*N*_b_-desmethyl-*meso*-chimonanthine (**44**)	*P. lyciiflora*	[49]	[49]
Psychotriasine (**45**)	*P. calocarpa*	[43]	[43]
Psychohenin (**46**)	*P. henryi*	[47]	[47]
Compound (**47**)	*P. henryi*	[48]	[48]
Compound (**48**)	*P. henryi*	[48]	[48]
Glomerulatine A (**49**)	*P. glumerulata*	[51]	[51]
Glomerulatine B (**50**)	*P. glumerulata*	[51]	[51]
Glomerulatine C (**51**)	*P. glumerulata*	[51]	[51]
Hodgkinsine (**52**)	*P. colorata* *P. oleoides* *P. lyciiflora* *P. muscosa* *P. beccarioides* *P. rostrata*	[41,42,43,44,45,46]	[42]
Psychotrimine (**53**)	*P. rostrata*	[2]	[2]
Psychotripine (**54**)	*P. pilifera*	[52]	[52]
Quadrigemine A (**55**)	*P. forsteriana*	[53]	[53]
Quadrigemine B (**56**)	*P. forsteriana* *P. colorata* *P. rostrata*	[41,53]	[53]
Quadrigemine C (**57**)	*P. colorata* *P. oleoides*	[41,42,43,45,46,50,54]	[45]
Quadrigemine I (**58**)	*P. oleoides*	[49]	[49]
Psycholeine (**59**)	*P. oleoides*	[46,54]	[46]
Psychopentamine (**60**)	*P. rostrata*	[2]	[2]
Psychotridine (**61**)	*P. forsteriana* *P. oleoides* *P. colorata* *P. beccarioides*	[41,44,45,53]	[45]
Isopsychotridine C (**62**)	*P. forsteriana*	[53,55]	[55]
Isopsychotridine B (**63**)	*P. oleoides*	[49,50]	[45]
Oleoidine (**64**)	*P. oleoides*	[49]	[49]
Caledonine (**65**)	*P. oleoides*	[49]	[49]

**Table 4:** ^13^C-NMR data of pyrrolidinoindoline alkaloids from *Psychotria* species.

**Carbons**	**Compounds/*δ*_C_ (ppm)**								
	**39 ^II^**	**40 ^II^**	**41 ^ns^**	**42 ^II^**	**43 ^II^**	**44 ^II^**	**45 ^I^**	**46 ^I^**	**47 ^II^**
**C**									
**3**	-	-	-	-	-	-	112.7	110.0	112.3
**3a**	64.7	63.6	37.8	36.8	48.9	62.8	-	-	-
**4a**	133.7	128.3	127.0	125.9	125.6	132.2	130.4	130.5	130.0
**7a**	152.5	150.5	145.3	146.2	145.8	151.7	137.7	138.0	135.0
**8a**	-	-	-	-	165.0	-	-	-	-
**3′a**	64.7	63.6	37.8	36.8	48.9	63.9	79.4	77.8	75.3
**4′a**	133.7	128.3	127.0	125.9	125.6	130.0	131.3	131.3	128.9
**7′a**	152.5	150.5	145.3	146.2	145.8	150.3	152.5	152.7	152.4
**8′a**	-	-	-	-	165.0	-	-	-	-
**CH**									
**2**	-	-	-	-	-	-	125.0	126.1	123.4
**4**	125.2	124.9	118.3	117.1	123.0	123.9	124.7	119.5	119.0
**5**	119.2	122.3	122.2 ^b^	122.1	118.5	119.9	119.3	118.8 ^e^	119.1 ^f^
**6**	128.9	129.9	127.7	127.3	128.2	128.2	130.7	123.0	121.4
**7**	109.5	110.5	112.9	112.8	123.9	109.1	120.1	117.4	112.8
**8a**	83.9	84.6	71.7	71.82	-	79.3	-	-	-
**4′**	125.2	124.9	118.3	117.1	123.0	124.4	112.2	124.9	126.4
**5′**	119.2	119.8	125.2	125.2	121.9	117.9	122.4	119.8	118.7
**6′**	128.9	129.9	127.7	127.3	128.2	128.4	119.6	130.9	130.2
**7′**	109.5	110.5	112.9	112.8	123.9	108.2	110.0	110.4	108.8
**8′a**	83.9	84.6	71.7	71.82	-	82.4	87.0	87.3	86.6
**CH_2_**									
**2**	53.1	52.4	46.9	47.3	48.5	44.9	-	-	-
**3**	36.4	33.2	34.9	32.5	29.9	35.3	-	-	-
**2′**	53.1	52.4	46.9	47.4	48.5	51.8	52.0	52.3	53.6
**3′**	36.4	33.2	34.9	32.5	29.9	38.1	39.9	40.0	37.7
**2″**	-	-	-	-	-	-	-	-	69.1
**CH_3_**									
**Me-N^1^-**	nd	33.8	46.9	43.4	31.1	-	36.3	33.9	40.6
**MeN^1′^-**	nd	33.8	46.9	43.4	31.1	35.12	35.7	36.4	37.1
**Carbons**	**Compounds/*δ*_C_ (ppm)**								
	**48 ^II^**	**49 ^III^**	**50 ^III^**	**51 ^III^**	**52 ^II^**	**53 ^II^**	**54 ^I + II^**	55 ^II,^*	56 ^II,^*
**C**									
**2**	129.6	-	-	-	-	-	-	-	-
**3**	109.4	-	-	-	-	114.9	-	-	-
**3a**	-	49.1	48.6	49.2	62.8	-	69.1	60.9 ^c^	60.1 ^c^
**4a**	128.0	126.4	126.1 ^a^	129.5	131.7	128.3	133.8	132.3 ^d^	133.2 ^e^
**7a**	137.4	177.3	147.1	148.6	150.8	136.1	152.2	150.9 ^h^	150.6 ^h^
**8a**	-	165.1	164.7	166.5	-	-	106.9	-	-
**3′a**	76.7	49.1	48.6	45.3	63.0	76.7	37.0	63.2 ^j^	63.9 ^i^
**4′a**	130.5	126.4	125.4 ^a^	122.3	132.3	132.0	122.0	132.4 ^d^	132.9 ^e^
**7′**	-	-	-	-	-	121.5	130.9	108.9 ^g^	-
**8′a**	-	165.1	164.7	-	-	-	-	-	-
**3″**	-	-	-	-	-	112.5	-	-	-
**3″a**	-	-	-	-	60.0	-	38.4	62.9 ^j^	63.3 ^i^
**8″**	-	-	-	-	-	25.7 ^b^	68.0	-	-
**9″**	-	-	-	-	-	52.0	-	-	-
**4″a**	-	-	-	-	131.7	129.8	122.3	132.6 ^d^	-
**7″a**	-	-	-	-	151.1	136.1	144.4	-	-
**3‴a**	-	-	-	-	-	-	-	60.8 ^c^	60.9 ^c^
**CH**									
**2**	-	-	-	-	-	126.0	-	-	-
**4**	117.9	123.7	120.9	123.0	126.4	119.4 ^a^	122.9	-	125.9 ^d^
**5**	119.2	122.3	122.2 ^b^	122.1	118.5	119.9	119.3	118.8 ^e^	119.1 ^f^
**6**	121.3	128.9	129.0 ^c^	128.8	127.9	122.4	128.2	127.9 ^f^	128.0 ^g^
**7**	112.1	125.0	125.2 ^d^	125.2	109.0	111.2	107.7	109.0 ^g^	108.9
**8a**	-	-	-	-	86.4	-	-	86.9 ^i^	85.9 ^i^
**4′**	124.5	123.7	124.0 ^d^	117.5	121.9	123.7	123.7	122.5	125.1 ^d^
**5′**	119.0	122.3	122.6 ^b^	124.9	116.8	119.3 ^a^	122.0	116.3 ^k^	118.3 ^f^
**6′**	129.6	128.9	128.8 ^c^	127.1	126.0	127.3	121.1	125.4	127.8 ^g^
**7′**	108.9	125.0	124.4 ^d^	114.4	-	-	-	-	-
**8′a**	86.5	-	-	76.5	81.7	86.1	69.7	86.1 ^i^	83.3 ^j^
**2″**	-	-	-	-	-	124.3	-	-	-
**4″**	-	-	-	-	124.2	119.3 ^a^	125.4	-	-
**5″**	-	-	-	-	117.5	119.3 ^a^	117.8	118.7 ^e^	117.2 ^f^
**6″**	-	-	-	-	127.4	121.7	127.6	-	-
**7″**	-	-	-	-	108.1	112.2	112.5	-	-
**8″a**	-	-	-	-	82.3	-	69.4	-	82.3 ^j^
**8‴a**	-	-	-	-	-	-	-	-	87.1 ^i^
**5‴**	-	-	-	-	-	-	-	116.2 ^k^	116.8 ^f^
**6‴**	-	-	-	-	-	-	-	126.4 ^f^	-
**CH_2_**									
**2**	-	48.2	48.1	48.5	51.7	-	54.9	52.6 ^a^	52.3 ^a^
**3**	-	30.3	30.3	31.7	37.6	-	36.3	38.8 ^b^	38.5 ^b^
**2′**	51.2	48.2	48.1	50.5	51.9	51.7	42.3	52.5 ^a^	52.2 ^a^
**3′**	40.6	30.3	30.3	34.0	36.7	39.1	33.1	38.7 ^b^	36.6 ^b^
**2″**	-	-	-	-	51.9	-	45.9	52.2 ^a^	-
**3″**	-	-	-	-	38.0	-	33.7	38.5 ^b^	-
**3‴**	-	-	-	-	-	-	-	36.6 ^b^	-
**CH_3_**									
**Me-N^1^-**	44.8	30.9	30.8	30.7	35.2	36.3	36.4	35.7 ^l^	35.8 ^k^
**MeN^1′^-**	36.1	30.9	-	36.6	35.0	36.4	-	35.5 ^l^	35.7 ^k^
**Me-N^1″^-**	-	-	-	-	35.1	36.4	41.8	35.0^l^	35.6 ^k^
**Me-N^1‴^-**	-	-	-	-	-	-	-	-	35.2 ^k^
**Carbons**	**Compounds/*δ*_C_ (ppm)**								
	57 ^II,^*	58 ^II,^*	59 ^II,^*	**60 ^II^**	61 ^II,^*	62 ^II,^*	63 ^II,^*	64 ^II,^*	65 ^II,^*
**C**									
**3a**	60.6	60.0	59.6 ^b^	61.1	60.1 ^a^	60.9 ^c^	63.0 ^a^	60.4 ^c^	60.0 ^c^
**4a**	-	132.0	132.4 ^c^	132.9 ^b^	-	132.7 ^d^	-	132.8	132.1 ^e^
**7a**	-	-	-	152.8	-	150.6 ^f^	-	150.7 ^e^	150.5 ^f^
**3′a**	62.6	63.0	37.5 ^f^	63.1	62.9	63.7 ^h^	63.3 ^a^	63.3 ^c^	63.0
**4′a**	-	-	-	132.8 ^b^	-	132.0 ^d^	-	-	132.4 ^e^
**7′**	-	110.0 ^c^	-	123.8	-	-	-	-	108.8
**7′a**	-	-	-	151.0	-	148.9 ^f^	-	150.3 ^e^	148.9 ^f^
**5″**	-	-	-	136.2	-	117.1	-	-	-
**3″a**	62.6	-	38.0 ^f^	64.2	62.9	63.2 ^h^	59.8 ^c^	60.9 ^c^	-
**4″a**	-	-	-	132.6	-	-	-	-	-
**7″a**	-	-	-	149.8	-	-	-	-	148.6
**3‴a**	60.6	-	60.6 ^b^	62.3	60.6 ^a^	60.1 ^c^	59.8 ^c^	-	60.5 ^c^
**4‴a**	-	-	133.8 ^c^	138.6	-	-	-	-	-
**7‴**	-	-	-	120.4	-	-	-	-	-
**7‴a**	-	-	-	144.7	-	-	-	-	-
**3″″**	-	-	-	114.5	-	-	-	-	-
**3″″a**	-	-	-	128.3	60.8 ^a^	-	60.7 ^c^	-	-
**4″″a**	-	-	-		-	-	-	-	-
**CH**									
**4**	-	126.0	-	126.9	-	123.6	-	126.1 ^d^	125.3 ^d^
**4′**	-	124.0	-	122.1	-	122.2	-	124.1	125.2 ^d^
**5**	-	117.0 ^b^	-	118.8	-	119.1 ^e^	-	116.3	118.9
**6**	-	129.5	-	128.0	-	128.2	-	128.7	128.1
**7**	-	109.0 ^c^	-	110.5	-	109.0	-	109.3	107.7
**8a**	85.8 ^a^	88.0 ^d^	88.5 ^d^	87.3	86.0 ^b^	87.2 ^g^	81.8 ^b^	86.6	86.9
**5′**	-	118.5 ^b^	-	116.2	-	118.4 ^e^	-	119.4	117.3
**6′**	-	127.5	-	126.5	-	126.1	-	126.2	125.3
**8′a**	82.3	83.0 ^d^	74.0 ^g^	82.4	82.6 ^c^	85.8 ^g^	86.8 ^b^	82.8	86.0
**4″**	-	-	-	121.4	-	-	-	125.7 ^d^	124.1
**5″**	-	119.0 ^b^	-	-	-	117.1	-	-	-
**6″**	-	-	-	126.1	-	125.4	-	128.4	-
**7″**	-	-	-	108.5	-	-	-	-	-
**8″a**	82.3	-	72.0 ^g^	83.4	82.3 ^c^	83.1	86.8 ^d^	83.0	-
**4‴**	-	-	-	123.3	-	-	-	122.7	123.6
**5‴**	-	119.5 ^b^	-	118.8	-	-	-	-	-
**6‴**	-	128.5	-	125.1	-	-	-	-	-
**7‴**	-	-	-	120.4	-	-	-	-	-
**8‴a**	86.7 ^a^	-	87.5 ^d^	88.4	86.9 ^b^	82.1	85.5 ^d^	-	-
**2″″**	-	-	-	126.1	-	-	-	-	-
**4″″**	-	-	-	119.3	-	-	-	125.7	123.2
**5″″**	-	-	-	111.2	-	-	-	-	-
**6″″**	-	-	-	122.3	-	-	-	-	-
**7″″**	-	-	-	119.7	-	-	-	-	-
**8″″a**	-	-	-	-	85.1 ^b^	-	84.8 ^d^	-	-
**CH_2_**	-	-	-	-	-	-	-	-	-
**2**	-	53.0	47.17 ^a^	52.7 ^a^	-	52.5 ^a^	-	52 ^a^	52.1 ^a^
**3**	-	38.0 ^a^	-	37.8	-	38.8 ^b^	-	38.7 ^b^	38.3 ^d^
**8**		-	-	-	-	-	-	-	-
**9**		-	-	-	-	-	-	-	-
**2′**		-	-	52.7 ^a^	-	52.0 ^a^	-	52.8 ^a^	52.4 ^a^
**3′**		39.0 ^a^	32.8 ^e^	35.8	-	38.5 ^b^	-	38.7 ^b^	38.6 ^b^
**2″**		-	-	52.6^a^	-	-	-	52.9 ^a^	-
**3″**		-	32.4 ^e^	37.2	-	-	-	-	-
**2‴**		-	48.0 ^a^	52.5^a^	-	-	-	-	-
**3‴**		-	-	39.2	-	-	-	-	-
**3″″**		-	-	114.5	-	-	-	-	-
**CH_3_**		-	-	-	-	-	-	-	-
**Me-N^1^-**		36.0	36.1 ^h^	34.8	-	35.6 ^i^	-	35.7	35.4 ^g^
**Me-N^1′^-**		-	42.6 ^i^	35.3	-	35.1 ^i^	-	-	35.6 ^g^
**Me-N^1″^-**		-	42.6 ^i^	35.8	-	-	-	-	-
**Me-N^1‴^-**		-	36.1 ^h^	35.7	-	-	-	-	-
**Me-N^1″″^-**		-	-	36.5	-	-	-	-	-

^I^ CD_3_OD, ^II^ CDCl_3_ e ^III^ benzene-*d*_6_, ^ns^ not specified; letters indicate signals that may be interchanged, nd = not detected; * indicates cases for which there was no complete detailed attribution of carbon signals.

**Table 5:** Benzoquinolizidine Alkaloids from *P. klugii*.

Compound	Reference	^13^C-NMR Data
Klugine (**66**)	[7]	[7]
7’-*O*-Demethylisocephaeline (**67**)	[7]	[7]
Cephaeline (**68**)	[7]	[56]
Isocephaeline (**69**)	[7]	[56]
7-*O*-Methylipecoside (**70**)	[7]	[57]

**Table 6:** ^13^C-NMR Data of Benzoquinolizidine Alkaloids from *P. klugii*.

Carbons	Compound/*δ*_C_ (ppm)				
	66 ^ns^	67 ^ns^	68 ^II^	69 ^II^	70 ^I^
**C**					
**6**	-	-	-	-	146.5 ^a^
**7**	-	-	-	-	147.8 ^a^
**9**	146.5 ^a^	146.8	147.2 ^a^	147.2 ^a^	-
**10**	147.8 ^b^	148.0	147.5 ^a^	147.4 ^a^	-
**4a**	-	-	-	-	126.9
**7a**	127.8	126.9	126.8	126.5	-
**8a**	-	-	-	-	130.2
**11a**	129.7	127.9	130.1	129.9	-
**1′**	79.5	-	-	-	-
**4′**	-	-	-	-	111.7
**4’a**	127.7	123.2	127.6	127.9	-
**6′**	146.4 ^a^	145.6	143.9 ^b^	144.0	-
**7’**					
**11’**	-	-	-	-	169.2
**8’a**	129.7	126.0	131.1	131.0	-
**CH**					
**1**	-	-	-	-	50.6
**2**					
**3**	42.5	41.3	41.7	61.5	-
**5**	-	-	-	-	116.2
**8**	116.2	112.1	111.5	111.4	111.1
**11**	109.7	109.0	108.6	108.2	-
**11b**	63.8	62.7	62.4	62.8	-
**1′**	-	53.6	51.9	55.3	98.7
**3′**	-	-	-	-	153.1
**4′**	28.5	27.6	29.0	29.3	-
**5′**	116.4	115.2	114.7	114.8	27.5
**8′**	110.0	113.2	108.4	108.6	136.3
**9′**	-	-	-	-	45.1
**CH_2_**					
**1**	40.6	36.9	36.9	39.3	-
**3**	-	-	-	-	36.1
**4**	62.2	61.6	61.3	52.6	29.1
**6**	53.3	51.9	52.3	52.6	-
**7**	29.3	25.3	29.2	29.1	-
**12**	24.4	23.3	23.6	24.0	-
**14**	37.0	38.0	40.9	40.4	-
**3′**	41.0	39.5	40.1	41.4	-
**4′**	28.5	27.6	29.0	29.3	-
**6′**	-	-	-	-	41.1
**10′**	-	-	-	-	120.1
**CH_3_**	11.5	10.1	11.2	11.3	-
**13**	11.5	10.1	11.2	11.3	-
**Me^7^-O-**	-	-	-		56.5
**Me^9^-O-**	-	55.4 ^d^	55.8 ^e^	55.8 ^f^	-
**Me^10^-O-**	56.8 ^c^	55.8 ^d^	56.0 ^e^	56.0 ^f^	-
**Me^7′^-O-**	56.6 ^c^	-	56.3 ^e^	56.0 ^f^	-
**Glucose**					
**1″**	-	-	-	-	100.5
**2″**	-	-	-	-	74.8
**3″**	-	-	-	-	78.2 ^b^
**4″**	-	-	-	-	71.5
**5″**	-	-	-	-	78.3 ^b^
**6″**	-	-	-	-	62.7
**CO_2_Me**					51.7

^I^ CD_3_OD, ^II^ CDCl_3_ e ^ns^ not specified; letters indicate signals that may be interchanged.
